# Crafts as a key factor in local development: Bibliometric analysis

**DOI:** 10.1016/j.heliyon.2023.e13039

**Published:** 2023-01-16

**Authors:** David Fernández Bellver, M. Belén Prados-Peña, Ana M. García-López, Valentín Molina-Moreno

**Affiliations:** a University of Granada; Department of Management-1, Faculty of Economics and Business Administration, Campus de Cartuja, s/n 18071, Granada, Spain; b University of Granada; Department of Marketing and Market Research; Faculty of Economics and Business Administration, Campus de Cartuja, s/n 18071, Granada, Spain; c University of Granada, Drawing Department, Faculty of Fine Arts, Avd. Andalucia S/N, Granada, Spain; d University of Granada; Department of Management-1, Faculty of Economics and Business Administration, Campus de Cartuja, s/n 18071, Granada, Spain

**Keywords:** Crafts, Local development, Bibliometric analysis, Sustainable development

## Abstract

The role of crafts on a global level has accrued importance at present, both for developing countries and for rural development in general. Governments and institutions are increasingly trying to promote rural development to fight against the flight of the population from rural areas. Crafts is considered an important tool for local economic development and job creation. The goal of this study is to use bibliometric analysis to analyze the advances in research in the field of crafts and their influence on the development of rural communities. It also aims to identify the main lines of research that are currently being addressed as future trends. This analysis has provided a global, systematic and visual overview of the 1379 studies related to the role of crafts in the development of rural areas, published from 1954, year in which the first publication appeared, up to 2021. Growth trends have been identified in the number of articles published, magazines, authors, institutions and most productive countries. Results have shown that the most popular lines of research on this subject are those in which crafts are considered a source of income for local communities, particularly linked to tourism, job creation and sustainability in the first place; followed by research on the demographic and economic effects of new craft products and processes on rural areas; and those that consider crafts as a factor to mitigate poverty in the rural world. Therefore, the concept of handicrafts as a source of livelihood for poor rural regions is primarily emphasized.

## 1 Introduction

In recent decades, rural development has involved a growing effort by governments, as shown by initiatives such as the Craft Project or Interreg Europe's Craftscode such as the European Agricultural Fund for Rural Development (EAFRD), with a budget of 95.5 billion euros for the period 2021–2027 [1].

In 2018, more than 30% of the EU's population lived in rural areas, occupying an area of 83% of its total surface [2]. According to the mentioned report, the population in rural areas has the highest age rates in the EU (over 50 years of age); showing the highest percentage of the population at risk of social exclusion and the decrease in the rural active population. In 2018, the average GDP per capita in rural regions was only 75% of the EU average [2]. Although in 2020, 43.84% of the world's population lived in rural areas [3], there is a clear rural demographic decline [4]. European projects such as ESCAPE [4] or the Plan of Measures to Combat the Demographic Challenge promoted by the Spanish Government's Ministry for the Ecological Transition and the Demographic Challenge, with a budget of more than 10 billion euros, and the main goals of fighting against depopulation and ensuring social and territorial cohesion, show the involvement of institutions and governments in combating this decline. In addition, rural areas face not only demographic changes, aging population, and greater risk of exclusion, but also present a high risk of poverty [2]. It is interesting to note that “poverty still has an overwhelmingly rural face” [5].

Fighting against the flight of population from rural areas to cities and efforts to reduce territorial inequalities relies mainly in rural development. In this context, crafts are crucial to combat depopulation [6].

Furthermore, crafts have traditionally stood out in the rural world as a source of job creation [7]. In this sense, there are also European projects, such as the European Network of Rural Artisans, which defines crafts as “part of the economy, due to their capacity to generate employment, as an element of social cohesion in the territories and as a potential tourist resource and distinctive culture resource of the counties” [8]. Moreover, according to Richards creative tourism allows a bridge between local and global creativity to be established, a key aspect, according to this author, to encourage innovation and community development, being especially important for local communities. Therefore, the emergence of tourist crafts in rural areas, provided by local people, is a source of subsistence or economic well-being that is linked to growing tourism [9] being craft itself, most of the time, a motivation for tourism development [10]. Many villages base their income on the sale of regional and local handicraft products to tourists ([[11], [12], [13], [14]]).

Furthermore, the importance of the impact of crafts on GDP must be highlighted, especially in developing countries, in which activities such as artisanal mining has a direct impact on employment and represent a large contribution to national GDP ([7,15]).

Crafts contribute to job creation with a significant role in rural development ([[16], [17], [18]]). It was possible to verify the growing concern both at the international level and at the national level due to the existing inequality at the territorial level between urban areas and increasingly depopulated rural areas.

With regard to the environmental aspects, it was possible to demonstrate that craftsmanship can achieve the best possible work performance without damaging the environment ([19]), conserving biodiversity and helping poorer communities to access higher incomes [20]. Arts and crafts can therefore be a sustainable business [21]. In this sense, crafts are a cleaner, more sustainable and ecological activity than industrial activities, such as, for example, the production of craft beer [22]; fashion designs [23]; *batik* production in Indonesia [24]; papermaking [25]; jewelry processes [26]; or the use of wood waste to treat gray water [27].

Having analyzed all these factors, it is also important to look at whether this interest is reflected in the academic community.

### 1.1 Objetives

This research aims to discover the studies that have been published to date on crafts and their influence on the development of rural areas.

It develops a bibliometric analysis with the aim of highlighting the importance of crafts as a factor in the development of rural areas, as well as its possible contributions within the framework of sustainable development, the promotion of employment, the movement of the population out of rural areas, the development of tourism and the advantages that crafts can offer compared to other sectors. Furthermore, it is considered of interest in the study to outline ideas of circular economy in the field of handicrafts.

## 2 Literature review

### 2.1 Crafts

Craft production can be defined as the production of “an item that fulfils a function, requires the use of hands to create and uses materials identified as natural” ([28]), pp. 140). Craft is a highly sustainable activity [29]; contributes positively to job creation [17]; and tourism development. Tourists have expressed their interest in the local traditional crafts of the areas they visit, promoting heritage conservation and restoration activities, employment creation and improvement incomes for the local population living near historic environments [30].

Besides, it can also encourage a better use of materials and processes an environmentally conscious attitude as expressed in the following extract:

*“The importance of inherited crafts and traditions presented in physical and moral heritage for every region was evident. When the built environment is designed in consistency with the existing heritage and people traditions, it facilitates the establishment of craftsmanship and the mental image of the place. It helps fulfilling the needs of the residents and positively affects their behavior with the environment as well as the visitors”* ([31], pp. 22)

On the other hand, crafts often have local identity as a differentiating element, due to knowledge of the context in which craft is developed, cultural response, holistic practices or production the local community to which the handicrafts belong [32]. These authors identify tradition as a characteristic of craftsmanship, recognizing the product, the manufacturing process and the history or ideas that surround it as elements that have passed on from generations to generations, adding a particular value to each work of art.

### 2.2 Rural development

Rural development, as a measure to avoid depopulation through migration to urban areas, faces the main challenge of providing rural people with economic resources (in terms of income, as opposed to urban areas with more and better employment and entrepreneurship opportunities) ([12,30]); technological improvements and resources, such as direct access to electricity, internet and water [33] or transport, both passenger and goods accessibility, sufficient to improve the quality of life of the inhabitants of a rural region [34], thus helping to avoid the well-known “rural exodus".

Due to the importance of rural development, the Cork Declaration took place in 1996, as a result of the European Conference on Rural Development; in recent decades, millions of euros have been invested by governments to alleviate this phenomenon.

One of the objectives of rural development is poverty alleviation. Several aspects need to be taken into consideration when fighting poverty such as boosting the agricultural engine, analyzing the viability of small farms, the rural non-farm economy, and implementing appropriate government measures [35]. Energy access can contribute to poverty reduction. The new technologies are also helping the agricultural sector to save time and resources, improve communications and Internet access, lengthen the working day thanks to more and better lighting, among other things [33]. Regarding the internet, access to information as a source of knowledge and communication possibilities are essential to empower rural communities [36], and e-commerce possibilities can help increase sales.

### 2.3 Crafts and rural development

Craft activity clearly favours rural development ([[7], [19], [37]]; among others). On the one hand, according to Richards and Sanchis, Serrano and Köster, crafts have a direct effect on employment in rural areas. In fact, several case studies have observed how crafts have been the protagonist of a great expansion in the 21st century due to the promotion of employment, in addition to the creation of new links between companies and the development of local trade [38]. On the other hand, and according to Ref. [19], crafts are a source of income for local communities and a sustainable activity. In this context, it is not surprising that crafts are key actions in governments and public administration's policies in fighting against rural demographic decline ([4,39,40]). There are numerous examples of this, such as the revitalization of local businesses and communities through crafts and cultural heritage in Japan [11], the importance of crafts for rural development and the keys to their development in China and India ([12,41]), or rural markets, the key part of which are craft enterprises. These can be the basis for development, innovation, business networks and supply chains, functioning as an engine of money circulation in local UK economies [42].

Moreover, tourism is an important source of income for local communities worldwide ([9,10,42]); and, in particular, the sale of locally produced handicrafts ([[11], [12], [13], [14]]).

Given the importance of crafts in local development and the current problems in relation to rural areas, the aim of this research is to analyze the degree of interest that this topic has aroused in academic literature; besides, it aims to determine to what extent can crafts can be considered a productive sector that encourages the development of rural areas. In addition, we will try to identify the role of the circular economy and its influence in this field of research. It seeks to answer the following research questions to achieve valuable results:

Q1. What was the direction of this area of research since its inception?

Q2. What characteristics do the most important publications have?

Q3. Which countries, authors and institutions are the most important in this field of research?

Q4. Is there international cooperation? If so, how do countries, authors and institutions cooperate?

Q5. How have the lines of research evolved to date?

Q6. Within the scope of this study, what relevance and evolution does the concept of the circular economy have?

## 3 Methodology

To carry out this research, bibliometric analysis technique has been used. Bibliometric analysis or scientometrics is a technique that consists of the application of mathematical and statistical methods to carry out a quantitative study of bibliographic units ([[43], [44], [45]]), making it possible to understand the path and evolution of a certain subject area.

**Fig. 1:**
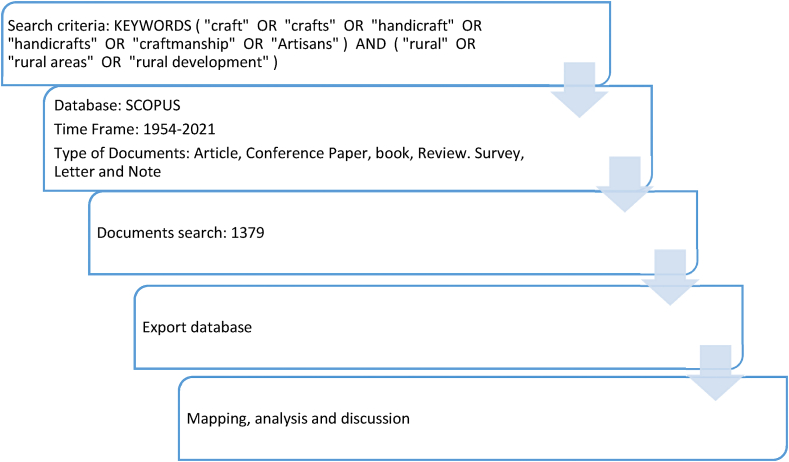
Methodology. Source: Author's own conception, based on Microsoft Word software.

The following steps are involved in this methodology, as shown in Fig. 1, and detailed below, in accordance with the structure of presentation of research methods followed by Cicea et al.:

a) Choosing the scientific database. First, the Scopus database was used for the selection of documents. The decision of using Scopus database in based on the largest volume of information regarding authors, countries and institutions provided ([18,46]). In addition, it contains the largest volume of articles and journals that meet the scientific quality requirements for peer review [47]. Its coverage is greater than that of the Web of Science [48] and it shows additional details of the publications [49]. Therefore, the Scopus platform was used to identify published academic works that would allow the contribution of sustainable crafts to the economic development of rural areas to be analyzed.
b) Creating a proper search syntax. Secondly, the search string was created considering keywords that link handicraft terms with rural development. After several tests in which different keywords were added and removed to find the best result, the final search resulted as follows: KEYWORDS (“craft” OR “crafts” OR “handicraft” OR “handicrafts” OR “craftmanship” OR “Artisans”) AND (“rural” OR “rural areas” OR “rural development”). This search acknowledges any publication that includes any of the terms included in the first parenthesis (craft terms in general) and in which, in addition, any of the terms in the second parenthesis (related to the rural world) appear.
c) Establishing the time span for the analysis. Regarding the time horizon, we felt that it was reasonable for the analysis to consider all the available years, to analyze the evolution of this line of research since its inception. The bibliographic search was carried out in February 2022 and 1379 documents were found.
d) Choosing the proper software for the Research. The VosViewer tool was used to develop visual analyses and diagrams of the information obtained. This tool is used to generate network maps of each variable, so that they can be grouped by clusters, establish relationship maps and process the terms included in the documents researched [50], thus showing the research topics that may be of most interest to the scientific community.

As mentioned above, Fig. 1 summarizes the methodological procedure described. In this way, the relationships between the publications were obtained, which allowed the conclusions of this research to be established and the trend of future lines of research based on this theme to be defined.

## 4 Results

The search string used by Scopus resulted in 1379 publications found, which were used to carry out the analyses that are subsequently developed. However, when evaluating and comparing journals, collecting the keywords and observing the evolution of the lines of research, only articles were considered, disregarding all other published documents for ease of comparison and understanding. Being only articles considered, the search yielded 1031 results. This was taken into account to ensure a higher scientific quality [48]. Thus, all documents were considered in the analyses related to scientific production, thematic areas, the productivity of publications of authors, countries, institutions, or international collaboration between them and only the articles were taken into account for the analyses of magazines and keywords. As for the time horizon, the entire available period was considered, excluding year 2022. The results show publications from 1954 up to the end of 2021.

### 4.1 Evolution of scientific production

**Fig. 2:**
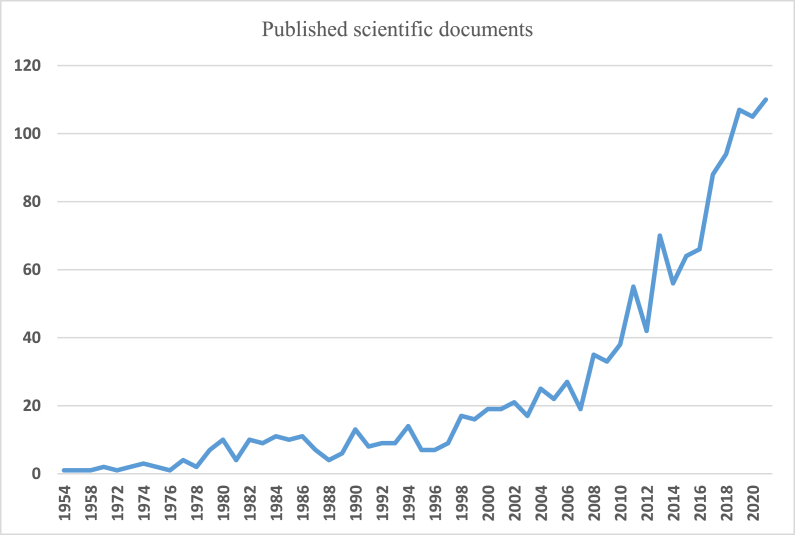
Evolution and trend of scientific production. Period 1954–2021. Source: Author's own conception, based on Microsoft Excel software.

Scientific production began with a publication in 1954, although there are years in which no publication related to the subject is found. Subsequently, production grows until a clear exponential trend can be seen. Fig. 2 shows the evolution of publications.

**Fig. 3:**
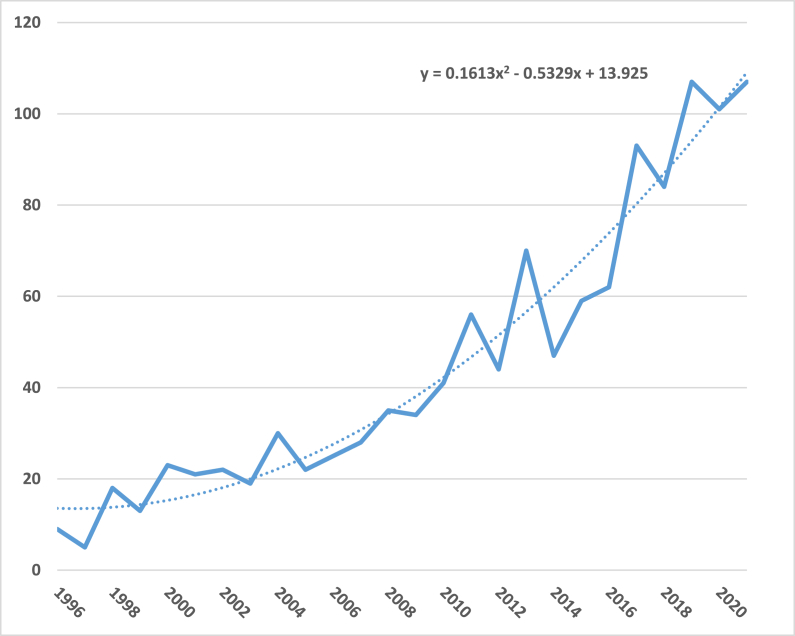
Annual evolution of scientific production. Period 1996–2021. Source: Author's own conception, based on Microsoft Excel software.

The Fig. 3 shows annual ecolution of scientific production since 1996 until 2021. The analysis focused on the period 1996–2021 for several reasons: on the one hand, the scarce scientific production, as well as its little importance; and on the other hand, in 1996, the European Conference on Rural Development culminated in the Cork Declaration, which was a new guide in rural development [51]. All this gave rise to a growing evolution, both in publications, institutions and participating countries as well as in the number of citations in the study area.

Table 1 presents a summary of the characteristics of scientific production in the field of crafts in the rural world.

**Table 1:** Characteristics of scientific production.

Year	Articles	Authors	Countries	Institutions	Citations	Journals	Citations per article	Average number of authors
1996	9	10	9	9	51	8	5.67	1.11
1997	5	5	3	4	44	5	8.8	1
1998	18	30	8	27	50	17	2.78	1.67
1999	13	28	9	15	38	13	2.92	2.15
2000	23	43	18	32	62	23	2.7	1.87
2001	21	33	15	26	63	21	3	1.57
2002	22	31	14	23	74	20	3.36	1.41
2003	19	34	12	27	105	19	5.53	1.79
2004	30	47	14	44	133	28	4.43	1.57
2005	22	38	20	29	137	20	6.23	1.73
2006	25	38	13	20	176	22	7.04	1.52
2007	28	50	19	44	201	28	7.18	1.79
2008	35	65	23	52	232	31	6.63	1.86
2009	34	66	22	48	260	32	7.65	1.94
2010	41	68	22	58	283	40	6.9	1.66
2011	56	144	29	95	321	56	5.73	2.57
2012	44	86	22	59	373	40	8.48	1.95
2013	70	145	33	94	419	67	5.99	2.07
2014	47	143	31	95	418	43	8.89	3.04
2015	59	136	26	114	476	59	8.07	2.31
2016	62	151	40	109	590	62	9.52	2.44
2017	93	186	36	128	693	84	7.45	2.00
2018	84	213	39	149	726	75	8.64	2.54
2019	107	274	50	193	785	87	7.34	2.56
2020	101	245	48	167	968	83	9.58	2.43
2021	107	318	42	222	1152	96	10.77	2.97
2001–2021	**1107**	**2511**	**570**	**1796**	**8585**	**1013**	**7.76**	**2.08**

Source: Author's own calculation, based on Microsoft Excel software.

A growing trend in the number of documents can be observed, showing the importance that this area of research has acquired in recent years. Greater growth is observed as of 2008, the year of the global financial crisis, which brought about important changes in rural areas, accentuating the segmentation between countries and regions and producing “massive cuts in the public budget,” giving rise to a worsening of employment in rural areas, especially in the most remote ones [52]. However, some authors such as [53] Giannakis and Bruggeman observe an increase in migration to rural areas as a consequence of the crisis, given that, according to them, rural areas cope better with economic crises than urban areas, with a better and greater response capacity [17].

### 4.2 Analysis of scientific production

This section shows the results of the analysis of the thematic areas, journals, authors and countries in which the selected works have been researched.

#### 4.2.1 Distribution of publications by thematic area

**Fig. 4:**
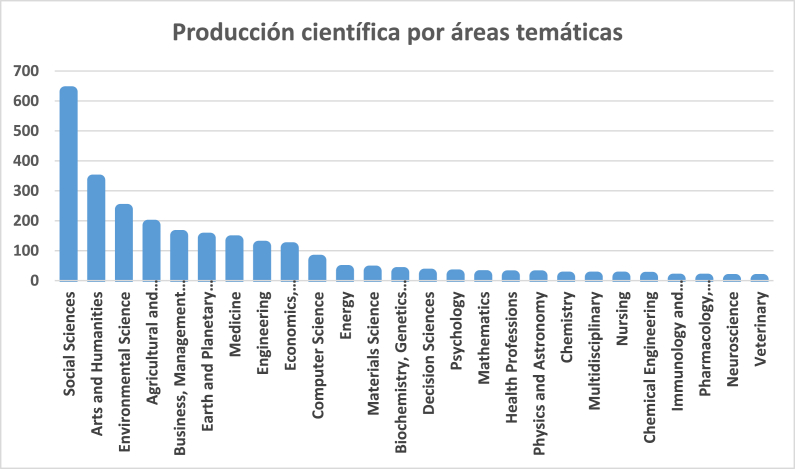
Scientific production by thematic areas. Source: Author's own conception, based on Microsoft Excel software.

Fig. 4 shows the scientific production by thematic areas. Of the 26 areas identified, Social Sciences is the one with the highest volume of documents (n = 631; 26.8%), almost double the quantity of the following, Arts and Humanities, (n = 336; 14.3%) and Environmental Sciences (n = 238; 10.1%). The fourth place is occupied by Agricultural Sciences and Biology (n = 185; 7.86%). These four areas concentrate 59% of the publications. This implies that the remaining 41% is distributed among 22 thematic areas, which shows the growing importance of this area of study not only in a thematic area but from different perspectives.

#### 4.2.2 Most cited articles

Table 2 shows the 10 most cited articles of the entire search carried out.

**Table 2:** The most cited articles.

Authors	Title	Year	Nº citations
Snell, K.D.M.	Annals of the labouring poor: social change and agrarian England 1660–1900.	1985	450
Epstein, S.R.	Craft guilds, apprenticeship, and technological change in preindustrial Europe	1998	300
Bhagavatula, S., Elfring, T., van Tilburg, A., van de Bunt, G.G.	How social and human capital influence opportunity recognition and resource mobilization in India's handloom industry	2010	219
Cecchini, S., Scott, C.	Can information and communications technology applications contribute to poverty reduction? Lessons from rural India	2003	193
Farsani, N.T., Coelho, C., Costa, C.	Geotourism and geoparks as novel strategies for socio-economic development in rural areas	2011	178
Long, P.T., Perdue, R.R.	The Economic Impact Of Rural Festivals And Special Events: Assessing The Spatial Distribution Of Expenditures	1990	151
Reijonen, H., Komppula, R.	Perception of success and its effect on small firm performance	2007	131
Godoy, R., Wilkie, D., Overman, H., Cubas, A., Cubas, G., Demmer, J., Mcsweeney, K., Brokaw, N.	Valuation of consumption and sale of forest goods from a Central American rain forest	2000	124
Gandini, G.C., Villa, E.	Analysis of the cultural value of local livestock breeds: A methodology	2003	114
Paxson, H.	Locating Value in Artisan Cheese: Reverse Engineering Terroir for New-World Landscapes	2010	97

Source: Author's own calculation, based on Microsoft Excel software.

The most cited article is by Snell [54]. This is a collection of essays studying economic and social change in workers and craftsmen in rural areas of the South of England and Wales from the mid-seventeenth century to the end of the nineteenth century. In a similar vein, Epstein [55] reports the important role of craft guilds during the Middle Ages and the role they played in pre-industrial times.

Other articles analyze the economic impact of different activities in rural areas, such as the article by Bhagavatula et al. [56] These authors highlight the importance of small businesses in rural areas as a key factor for the development of any country and specifically study the case of India and how social and human capital influences the mobilization of resources and the recognition of opportunities. For their part, Farsani et al. analyze the role of geoparks and geotourism and the importance of rural areas and the crafts sector for economic development in this context. Long et al. [57] analyze the economic impact of rural arts and crafts festivals on the local community and to what extent they contribute to the economic development of the community.

Other studies analyze some of the factors that help the development of small rural businesses, such as the work of Cecchini and Scott [58]. These authors show how ICT can help farmers and artisans to improve their quality of life. In a very similar line, Reijonen et al. analyze the perceptions and attitudes of entrepreneurs in small craft and rural tourism businesses, describing attitudes of success and growth.

Finally, other articles analyze the value of certain goods and their influence in the world of crafts and the rural world. Thus, Godoy et al. discusses the importance of the value of the tropical forest and how local residents receive a small part of its value; as well as the value of crafts, food, medicines or construction and their influence on the motivation to deforest. For their part, Gandini and Villa [59] analyze the cultural value of native livestock and its influence on life in rural areas. These authors also consider various factors, such as crafts or folklore, roles in the agricultural system and the landscape, etc., to assess the historical value of a local breed. Lastly, Paxson [60] deals with the craft of cheese, its influence in obtaining a superior quality, improving the environment, and achieving the economic revitalization of the rural world.

#### 4.2.3 Author productivity

This section presents the results of the productivity of the authors and international cooperation networks in the period analyzed. For the analysis of the cooperation network, it was taken into consideration whether they had at least two publications in crafts and rural development. Table 3 shows the authors who were most in the line of research.

**Table 3:** List of the 10 most productive authors.

Authors	A	CT	CT/A	Institution	P	1st A	UA	H
Banik, A.	4	12	3.00	International Management Institute, New Delhi	India	2005	2018	2
Baumann, V.H.	4	12	3.00	Institutul de Cercetǎri Eco-Muzeale, Tulcea	Rumanía	2009	2017	2
Bhaumik, P.K.	4	12	3.00	International Management Institute, New Delhi	India	2005	2018	2
Eyferth, J.	4	24	6.00	The University of Chicago	United States	2003	2006	3
Rogerson, C.M.	4	69	17.25	College of Business and Economics, Johannesburg	South Africa	1986	2019	4
Basak, J.	3	3	1.00	Indian Institute of Management Calcutta, Kolkata	India	2018	2020	1
Bhattacharjya, B.R.	3	9	3.00	Indian Institute of Technology Guwahati	India	2019	2020	2
Braedt, O.	3	33	11.00	Bundesforschungsanstalt fur Forst Und Holzwirtschaft, Hamburg	Germany	2000	2003	3
Braunegg, G.	3	26	8.67	ARENA Research for Sustainable Resources, Graz	Austria	2016	2020	2
Dang, T.D.	3	21	7.00	Australian Defence Force School of Languages	Australia	2013	2020	2

(A): Number of published documents (CT): total citations; (CT/A): average citations per article; (P): Country; 1st A: year of first publication; U A: year of last publication; (H): Index H in the line of research.

Source: Author's own calculation, based on Microsoft Excel software.

The most productive authors are Banik, A. from India, Baumann, V.H. from Romania, Bhaumik P.K., also from India, Eyferth J., from the United States and Rogerson C.M., from South Africa, with 4 publications in the area. Rogerson C.M. is the one with the highest H-index in the publications of the analyzed field [4]; they are followed by Eyferth J. and Braedt O., with an index of 3.

Concerning the dissemination of the results, measured according to the number of citations per article, the author with the highest average number of citations is Rogerson C.M., with an average of 17.25 citations, followed by Braedt O. and Braunegg G., with 11 and 8.67 average citations, respectively.

**Fig. 5:**
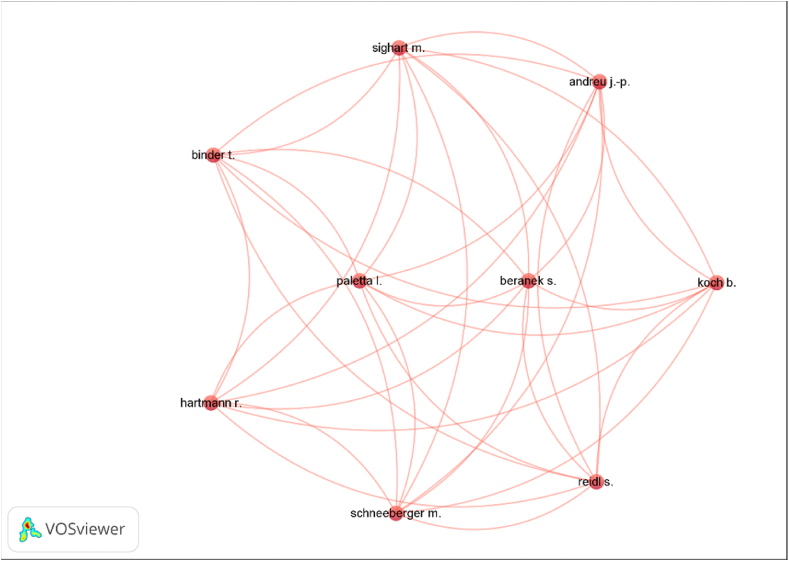
Map of international cooperation. Source: VosViewer v. 1.6.18.

Additionally, using the VosViewer tool, a map was generated showing the international cooperation networks of the co-authors that address the issue of crafts and their influence on rural development (see Fig. 5). For a total of 2767 authors, when selecting an interaction of at least 2 published articles, a total of 145 authors were obtained, of which only 9 formed a single cluster of international cooperation in the line of research.

It is noteworthy that none of the 10 most productive authors in the line of research belongs to an international cooperation network. Therefore, it can be stated that there is hardly any international cooperation network of co-authors in the line of research.

#### 4.2.4 Productivity of journals, institutions and countries

This section shows the productivity and cooperation of research in the area studied by journals, institutions and countries. Table 4 shows the 10 most productive journals.

**Table 4:** List of the 10 most productive journals.

Journal	A	TC	TC/A	H index articles	H index journal	SJR	C
Journal of Rural Studies	9	103	11.4	5	104	1.497 (Q1)	United Kingdom
Sustainability (Switzerland)	7	88	12.6	5	85	0.612 (Q2)	Switzerland
International Journal of Entrepreneurial Behaviour and Research	5	122	24.4	5	67	1.241 (Q1)	United Kingdom
Economic Botany	5	115	23.0	4	70	0.491 (Q2)	United States
World Archaeology	5	107	21.4	4	66	0.926 (Q1)	United Kingdom
World Development	5	75	15.0	5	175	2.386 (Q1)	United Kingdom
Journal of Peasant Studies	5	64	12.8	4	83	3.110 (Q1)	United Kingdom
Journal of Ethnobiology and Ethnomedicine	5	71	14.2	4	69	0.741 (Q1)	United Kingdom
Rural History	4	36	9.0	4	18	0.155 (Q3)	United Kingdom
Journal of Family History	3	45	15.0	3	22	0.169 (Q3)	United States

The Journal of Rural Studies, from the United Kingdom, is the one with the most publications on the subject, with 9 articles; it is followed by Sustainability, from Switzerland, with 7. However, the ones with the greatest circulation, due to the average number of citations per article, are the *International Journal of Entrepreneurial Behavior* and Research and the American *Economic Botany*, with an average number of citations per article of 24.4 and 23 respectively.

Concerning the H-index, the journal World Development with an H-index of 175 stands out from the others, followed by the *Journal of Rural Studies*, with an H-index of 104. Both journals belong to the United Kingdom.

Regarding the institutions, Table 5 shows the most productive institutions of the analyzed period.

**Table 5:** List of the 10 most productive institutions.

Institutions	C	A	CT	CT/A	H	IC (%)	CT/A	
							CI	NIC
University of KwaZulu-Natal	South Africa	10	34	3.4	4	10.0	4.0	3.3
University of Illinois Urbana-Champaign	United States	9	60	6.77	4	22.2	2.0	8.0
University of the Witwatersrand, Johannesburg	South Africa	9	139	15.4	6	22.2	6.0	18.1
Universidade Federal Rural de Pernambuco	Brasil	7	36	5.1	3	0.0	0.0	5.1
London School of Economics and Political Science	United Kingdom	7	539	77.0	5	57.1	52.3	110.0
Indian Institute of Technology Guwahati	India	6	11	1.8	2	33.3	1.0	2.3
The University of British Columbia	Canada	6	87	14.5	4	50.0	16.7	12.3
University of California, Los Angeles	United States	6	174	29.0	5	16.7	26.0	29.6
Københavns Universitet	Denmark	6	40	6.7	3	66.7	4.3	11.5
University of South Africa	South Africa	5	21	4.2	2	20.0	2.0	4.8

(C): Country; (A): Number of articles published (CT): total citations; (CT/A): average citations per article; (H): Index H in the line of research; (IC): Cooperation Index; (TC/A IC): Average number of citations with international cooperation; (TC/A NIC): Average number of citations without international cooperation.

Source: Author's own calculation, based on Microsoft Excel software.

The most productive Institution is the University of KwaZulu-Natal, in South Africa, with 10 publications in the area of study. They are followed by the University of Illinois Urbana-Champaign in the United States and the University of the Witwatersrand, Johannesburg, also in South Africa, with 9 publications each.

The institutions with a higher H-index are the University of the Witwatersrand, Johannesburg in South Africa (H-index of 6), followed by the London School of Economics and Political Science in the United Kingdom and the University of California, Los Angeles, in the United States, both with an H-index of 5. The institutions with the lowest H-index are the Indian Institute of Technology Guwahati, India and the University of South Africa, with an H-index of 2.

On the other hand, concerning the dissemination of results and the average number of citations of the publications in the line of research, the most cited institution is the London School of Economics and Political Science, with an average of 77 citations per article. The institution with the lowest average number of citations per article is the Indian Institute of Technology Guwahati with 1.83 citations per article.

Regarding the network of international cooperation between institutions, the Københavns Universitet in Germany is the institution with the highest rate of international cooperation, with 66.7% of its publications in cooperation. They are followed by the London School of Economics and Political Science and the University of British Columbia in Canada, with rates of 57.1% and 50%, respectively. The institutions that collaborate the least are the South African University of KwaZulu-Natal, from South Africa, with 10%, and the Universidade Federal Rural de Pernambuco, from Brazil, which stands out for not conducting any type of international cooperation.

**Fig. 6:**
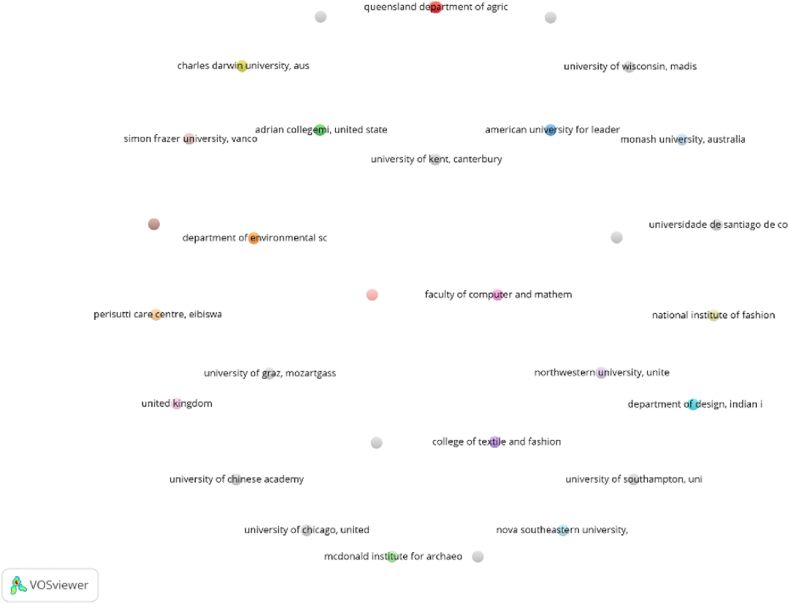
Map of international collaboration between institutions. Source: VosViewer v. 1.6.18.

Fig. 6 shows the international cooperation networks of the institutions. Of 1987 institutions identified, an interaction of at least 2 articles was selected and 31 international institutions were identified. The absence of connections denotes an absence of international collaboration between institutions in the line of research.

If, on the other hand, we analyze the productivity and collaboration between countries, Table 6 shows the countries that have had the most publications on the subject of study.

**Table 6:** Publications by country.

Country	A	CT	CT/A	H	NC	PPC	IC (%)	TC/A	
								IC	NIC
United States	197	2520	12.8	26	36	Canada, China, Australia, United Kingdom, India, Peru	16.8	19.4	11.5
India	155	871	5.2	12	8	Netherlands, United States	6.5	26.1	4.2
United Kingdom	127	1962	15.5	17	27	China, Canada, Ireland, Netherlands, United States	27.6	17.6	14.6
South Africa	50	486	9.7	12	11	Zimbabwe, Germany	24.0	12.3	8.9
Canada	44	592	13.5	13	17	United States, United Kingdom	36.4	20.8	9.3
China	43	301	7.0	9	9	United States, United Kingdom, Australia	32.6	14.4	3.4
Indonesia	38	63	1.7	5	4	Australia, Japan, Malaysia, United Kingdom	7.9	1.3	1.7
Italy	38	327	8.6	10	8	Brazil, Canada, Comoros, France, Germany	21.1	8.3	8.7
Brazil	36	248	6.9	9	5	Italy, Mexico, Mozambique, United Kingdom, United States	13.9	15.2	5.6
France	34	252	7.4	8	12	Morocco	23.5	10.5	6,5

(P): Country; (A): Number of publications; (CT): total citations; (CT/A): average citations per article; (H): Index H in the line of research; (NC): Publications without international collaboration; PPC: Main collaborating countries; (IC): Cooperation Index; (TC/A IC): Average number of citations with international cooperation; (TC/A NIC): Average number of citations without international cooperation.

Source: Author's own calculation, based on Microsoft Excel software.

In terms of productivity, the United States is the country with the most publications (197 publications), followed closely by India and the United Kingdom, with 155 and 127 publications, leaving a gap with the next country, South Africa, which has 50 publications in the area of study.

The United States is also the country that has achieved the greatest dissemination of its results, with by far the highest number of citations (a total of 2,520), followed by the United Kingdom with 1,962. However, the average number of citations, which is the best indicator of scientific quality, puts the UK in first place, with an average of 15.45 citations, followed by Canada and the US, with an average of 13.45 and 12.79, respectively.

Lastly, the H-Index places the United States firmly in the lead, with an H-Index of 26.

Regarding international collaboration, the United States and the United Kingdom are the countries with the highest number of international collaborators with 26 and 17 respectively. However, most of their articles are published with domestic authors, since their collaboration index is 16.8 and 27.6. Canada and China are the countries with the highest rate of collaboration, with rates of 36.4% and 32.6%, respectively. The least collaborative countries are India and Indonesia (6.5% and 7.9%, respectively). In this case, the two countries that collaborate the least are also the ones that have the fewest average citations.

**Fig. 7:**
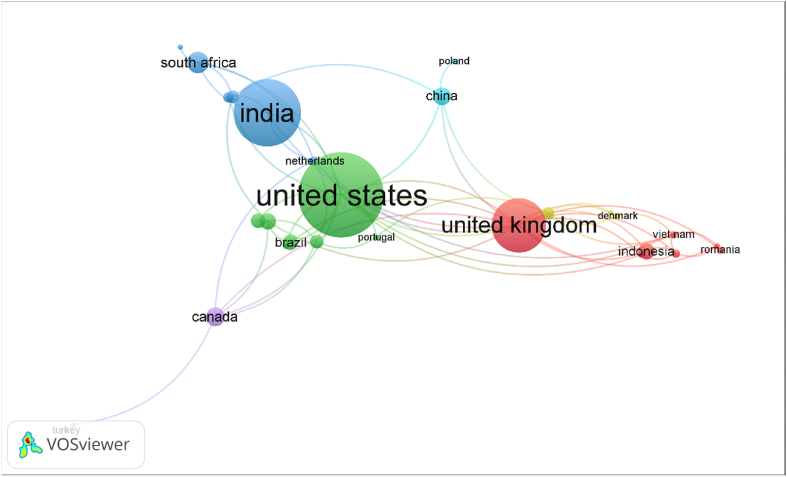
Map of international cooperation networks by country. Source: VosViewer v. 1.6.18.

Fig. 7 shows 6 different clusters, identified by colours, showing international collaboration networks. For a total of 142 identified countries, an interaction of at least ten published articles was applied, and 46 countries were identified, which made up 6 international cooperation networks.

The two most numerous clusters are made up of 7 countries each and are headed by the United States, on the one hand, and the United Kingdom, on the other.

Table 7 summarizes the clusters and their distribution by country. The cluster in which the United States is integrated is the one that surpasses all others in publications and citations, followed by the United Kingdom's cluster, with the China-Poland cluster in last place.

**Table 7:** Collaboration clusters between countries.

Clúster	Countries	Publications	Citations
1	United States, Brazil, France, Italy, Mexico, Portugal and Spain	365	3831
2	UK,Austria, Indonesia, Japan, Malaysia, Romania and Vietnam	246	2338
3	India, Germany, Iran, the Netherlands, Russia and South Africa	295	2092
4	Australia, Denmark and Sweden	53	533
5	Canada, Finland and Turkey	67	842
6	China and Poland	53	310

Source: Author's own calculation, based on Microsoft Excel software.

On the other hand, Table 8 shows the top ten countries and most cited publications in each country.

**Table 8:** Top ten countries and most important publications.

Country	Most cited publications	Author	Year	Cites
United States	Valuation of consumption and sale of forest goods from a Central American rain forest	Godoy et al.	2000	125
	Locating Value in Artisan Cheese: Reverse Engineering Terroir for New-World Landscapes	Paxson, H.	2010	106
	Tree and impervious cover in the United States	Nowak, D.J., Greenfield, E.J.	2012	99
	Filipina migrants in rural Japan and their professions of love	Faier, L.	2007	99
India	How social and human capital influence opportunity recognition and resource mobilization in India's handloom industry	Bhagavatula et al.	2010	239
	Prehistoric human colonization of India	Misra, V.N.	2001	137
	Body mass index: A measure of the nutritional status in Indian populations	Naidu, A.N., Rao, N.P.	1994	117
	Drudgery, Accidents and Injuries in Indian Agriculture	Nag, P.K., Nag, A.	2004	73
United Kingdom	Annals of the labouring poor: social change and agrarian England 1660-1900	Snell, K.D.M.	1985	467
	Craft guilds, apprenticeship, and technological change in preindustrial Europe	Epstein, S.R.	1998	309
	Can information and communications technology applications contribute to poverty reduction? Lessons from rural India	Cecchini, S., Scott, C.	2003	199
	Fracture trauma in a medieval British farming village	Judd, M.A., Roberts, C.A.	1999	94
South Africa	Tourist preferences for ecotourism in rural communities adjacent to Kruger National Park: A choice experiment approach	Chaminuka et al.	2012	95
	The traditional use of plants to manage candidiasis and related infections in Venda, South Africa	Masevhe, N.A., McGaw, L.J., Eloff, J.N.	2015	49
	Tourism, food, and culture: Community-based tourism, local food, and community development in mpondoland	Giampiccoli, A., Kalis, J.H.	2012	45
	Rural economy and livelihoods from the non-timber forest products trade. Compromising sustainability in southern Africa?	Dovie, D.B.K.	2003	40
Canada	Valuation of consumption and sale of forest goods from a Central American rain forest	Godoy et al.	2000	125
	Fracture trauma in a medieval British farming village	Judd, M.A., Roberts, C.A.	1999	94
	Rain forest ‘conservation-through-use’? Chambira palm fibre extraction and handicraft production in a land-constrained community, Peruvian Amazon	Coomes, O.T.	2004	38
	Commodity production and ethnic culture: Otavalo, northern Ecuador	Korovkin, T.	1998	38
China	Settlement patterns and development of social complexity in the Yiluo Region, North China	Liu et al.	2004	86
	Environmental impacts and embodied energy of construction methods and materials in low-income tropical housing	Hashemi, et al.	2015	39
	China's Hidden Agricultural Revolution, 1980–2010, in Historical and Comparative Perspective	Huang, P.C.C.	2016	30
	The Income Gap Between Urban and Rural Residents in China: Since 1978	Ma et al.	2018	25
Indonesia	The diversity of plant species, the types of plant uses and the estimate of carbon stock in agroforestry system in Harapan Makmur Village, Bengkulu, Indonesia	Wiryono, Puteri, V.N.U., Senoaji, G.	2016	13
	Hobby and business on trading birds: Case study in bird market of Sukahaji, Bandung, West Java and Splendid, Malang, East Java (Indonesia)	Iskandar, B.S., Iskandar, J., Partasasmita, R.	2019	12
	Species diversity and utilization of bamboo to support life's the community of Karangwangi village, Cidaun sub-district of Cianjur, Indonesia	Setiawati et al.	2017	9
	The use by local communities of plants from sesaot protected forest, West Nusa Tenggara, Indonesia	Hidayat, S.	2017	7
Italy	Analysis of the cultural value of local livestock breeds: A methodology	Gandini, G.C., Villa, E.	2003	118
	Sustainable rural development: The role of traditional activities in Central Italy	Gobattoni, et al.	2015	30
	Traditional uses of plants in a rural community of Mozambique and possible links with Miombo degradation and harvesting sustainability	Bruschi et al.	2014	29
	Increasing the value of spent grain from craft microbreweries for energy purposes	Sperandio et al.	2017	26
Brazil	Ethnobotany and effects of harvesting on the population ecology of Syngonanthus nitens (Bong.) Ruhland (Eriocaulaceae), a NTFP from Jalapao region, central Brazil	Schmidt, I.B., Figueiredo, I.B.,https://www.scopus.com/authid/detail.uri?origin=resultslist&authorId=6507887219&zone=Scariot,A	2007	75
	Ethnobotany in Cabo Delgado, mozambique: Use of medicinal plants	Matavele, J., Habib, M.	2000	37
	Experimental harvesting of the non-timber forest product Ischnosiphon polyphyllus in central Amazonia	Nakazono, E.M., Bruna, E.M., Mesquita, R.C.G.	2004	22
	Knowledge, Use, and Management of the Babassu Palm (Attalea speciosa Mart. ex Spreng) in the Araripe Region (Northeastern Brazil)	Almeida Campos et al.	2015	19
France	Traditional food and tourism: French tourist experience and food heritage in rural spaces	Bessiere, J., Tibere, L.	2013	75
	Beekeeping as a family artisan entrepreneurship business	Ramadani et al.	2019	45
	The French peasantry in the seventeenth century.	Goubert, P.	1986	38
	Physical activity patterns of rural Senegalese adolescent girls during the dry and rainy seasons measured by movement registration and direct observation methods	Bénéfice, E., Cames, C.	1999	36

Source: Author's own calculation, based on Microsoft Excel software

#### 4.2.5 Keyword analysis

The keyword analysis was carried out on the articles of the search for the area of crafts as a factor in the development of rural areas. In total, through the Scopus platform, 1033 articles were found from which the keywords would be extracted for the period 1954–2021.

**Fig. 8:**
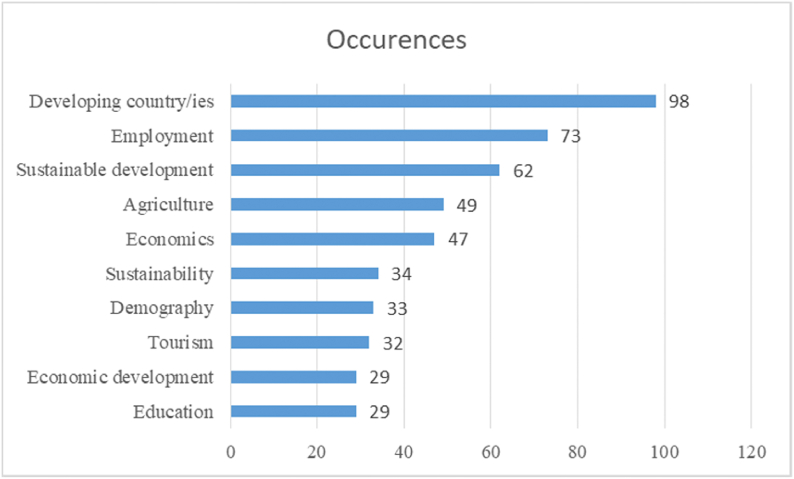
Ten more mentioned keywords Source: Author's own conception, based on Microsoft Excel software.

The tool used for the analysis was VosViewer. There was a total of 3232 keywords in the search. On the one hand, Fig. 8 shows the ten more mentioned keywords.

As Fig. 8 shows, the key word “Developing country”, also in its plural form, are the most used key words shown as result in this research, with a total of 98 appearances. Terms that show links between crafts and rural development follow the list of those that have been frequently used in the development of the research. These are “Employment”, “Sustainable development”, “Agriculture”, a term closely related to crafts in terms of the rural world, due to its relationship with the production of handicrafts for sale to tourists. These are followed by terms “Economics” and “Sustainability”, two very generic terms in this line of research, and “Demography”, another important concept within this work, since it approaches the problem of demographic decline. “Tourism”, a term strongly related to crafts from the point of view of its development and potential. Concluding with two different related and generic terms, namely “Economic development” and “Education”, being the last one included in this research in two aspects: on the one hand as a measure of development, on the one hand, and as ways of transmission of craft skills.

Table 9 shows for each of the main keywords identified, the publication containing them as well as the publication, its author/authors and the year of publication (see Table 9).

**Table 9:** Top ten keywords and most important publications.

Keyword	Author's publications	Author	Year
Developing contry/ies	Marketing rural products in India	Kashyap, P.	1991
	The contribution of non farm activities to rural employment promotion: Experience in Iran, India and Syria	Guha, S.	1974
	The growth of household industry in rural Wenzhou	Li Shi	1990
	An integrated ecosystem incorporating renewable energy leading to pollution reduction for sustainable development of craft villages in rural area: a case study at sedge mats village in Mekong Delta, Vietnam	Le et al.	2016
Employment	Mothers and Godmothers of Crafts: Female Leadership and the Imagination of India as a Crafts Nation, 1947–67	McGowan, A.	2021
	Challenges to sustainable growth of the micro-scale kuhila craft industry of India	Majumdar, P., Banerjee, S.	2017
	Application of online trading market in rural handicraft protection design strategy: - Take “Fang yuan” app handicraft exchange trading platform as an example	Dai, Y., Yang, D.	2020
	The contribution of non farm activities to rural employment promotion: Experience in Iran, India and Syria	Guha, S.	1974
Sustainable development	WECRAFT: A Platform for Networking Rural Craftsmen in Co-Production of Artisanal Crafts	Guerrieri, P.M., Comai, S., Fugini, M.	2021
	Challenges to sustainable growth of the micro-scale kuhila craft industry of India	Majumdar, P., Banerjee, S.	2017
	Environmental protection policies at craft villages in Hanoi in the context of sustainable development	Nguyen et al.	2021
	Environmental pollution in Vietnam's craft villages	Nguyen, T.L.	2020
Agriculture	WECRAFT: A Platform for Networking Rural Craftsmen in Co-Production of Artisanal Crafts	Guerrieri, P.M., Comai, S.,https://www.scopus.com/authid/detail.uri?origin=resultslist&authorId=35614277100&zone=	2021
	Challenges to sustainable growth of the micro-scale kuhila craft industry of India	Majumdar, P., Banerjee, S.	2017
	Environmental protection policies at craft villages in Hanoi in the context of sustainable development	Nguyen et al.	2021
	Upgradation of housing and amenities in rural areas	Chadha, P.S.	2005
Economics	Environmental pollution in Vietnam's craft villages	Nguyen, T.L.	2020
	Agrarian tourism as a factor in the socio-economic development of rural areas	Kolomyts et al.	2020
	Integrated rural development: commitment and policy-frame.	Patel, A.R.	1979
	Does the Informal Sector in Kenya Have Financial Potential to Sustainably Prepay for Health Care? Implications for Financing Universal Health Coverage in Low-Income Settings	Okungu, V.R., McIntyre, D.	2019
Sustainability	Challenges to sustainable growth of the micro-scale kuhila craft industry of India	Majumdar, P., Banerjee, S.	2017
	Crafting Sustainability? The Potential and Limits of Institutional Design in Managing Water Pollution from Vietnam's Craft Villages	Mahanty, S., Dang, T.D.	2013
	Sustainable and green design in villages of Rural Southwest China	Pitts, A., Gao, Y.	2017
	Assessing sustainable bamboo-based income generation using a value chain approach: Case study of nongboua village in Lao PDR	Lee et al.	2021
Demography	Integrated rural development: commitment and policy-frame.	Patel, A.R.	1979
	Assessing health impacts of an environmental pan-African development project: A migration perspective	Duboz et al.	2020
	Malignant lymphomas. Epidemiological review of 150 cases	Chillè et al.	1999
	Rural environmental attitudes	McBeth, M.K., Foster, R.H.	1994
Tourism	Preservation of Malaysian handicraft to support tourism development	Hassan et al.	2017
	Rural handicraft production in mpumalanga, south africa: Organization, problems and support needs	Rogerson, C.M., Sithole, P.M.	2001
	Tourism in rural areas: A case study of opportunities in the South Coast of KwaZulu-Natal	Mnguni, E.M., Mtapuri, O.,https://www.scopus.com/authid/detail.uri?origin=resultslist&authorId=38861553600&zone=	2020
	Employment and local development in rural environment | [Emploi et développement local en milieu rural]	Bandarra, N.J.	2000
Economic Development	Geotourism and geoparks as novel strategies for socio-economic development in rural areas	Farsani, N.T., Coelho, C., Costa, C.	2011
	Globalization and weavers' health in India-case study of Varanasi silk weavers	Zehra, M.	2016
	Art of Africa	Sleigh, M.	2005
	Integrated rural development: commitment and policy-frame.	Patel, A.R.	1979
Education	Upgradation of housing and amenities in rural areas	Chadha, P.S.	2005
	Educational farm as a new tourism product (The example of Podkarpackie province)	Mitura, T., Buczek-Kowalik, M.	2016
	The Effectiveness of Handicrafts on Anxiety Reduction among Hospitalised Children in Paediatric Ward of Dhulikhel Hospital	Ranamagar, B., Karki, S.	2021
	Educating and training craft textile producers	Perivoliotis, M.C.	2007

Source: Author's own calculation, based on Microsoft Excel software

Table 9 shows a summary of the most cited publications containing the ten most important keywords mentioned.

**Fig. 9:**
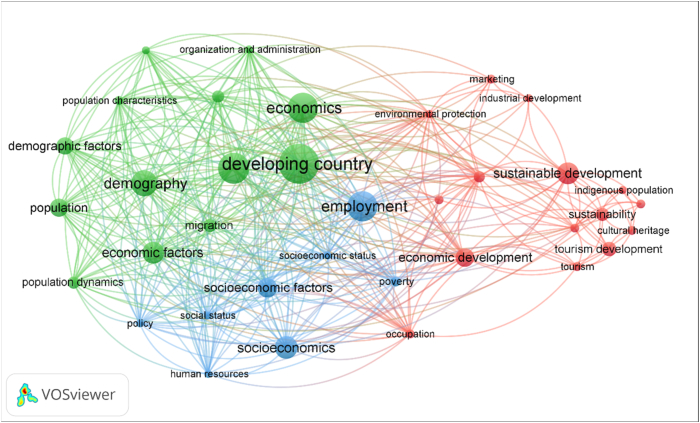
Network map of keywords on crafts and local development. Source: VosViewer v. 1.6.18.

Finally, a minimum of 10 matches were considered, obtaining a result of 90 keywords and the search was refined by eliminating those that included terms from the initial search, such as “rural” or “craft,” as well as other words that were discarded due to their nature as academic terms, research, or other unrelated topics. Thus, a final set of 39 clustered keywords was reached (see Fig. 9).

Fig. 9 shows the clusters of keywords included in the analysis, a total of 36. Three clusters are distinguished in red, green and blue. The largest cluster is the red cluster, made up of 15 items. It is followed by the green cluster with 13 items and finally, the blue cluster, with 8 items.

**Fig. 10:**
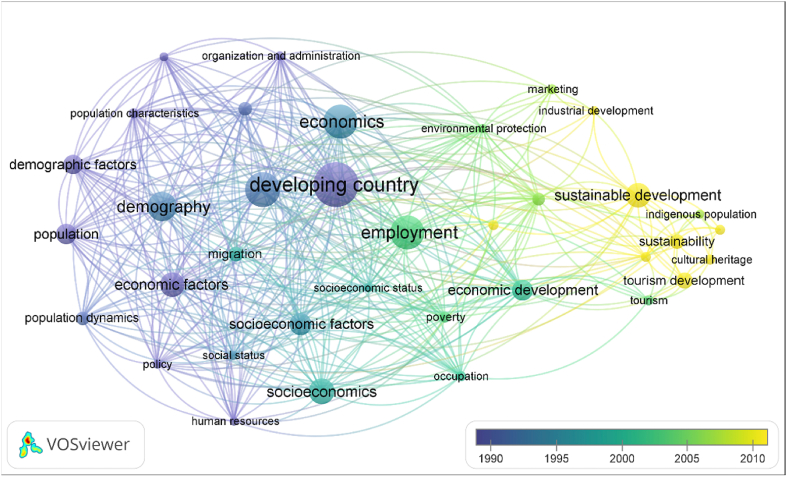
Network map of keywords of future lines of research in the study area. Source: VosViewer v. 1.6.18.

On the other hand, Fig. 10, concerning research trends, shows the most innovative lines of research in the keywords with lighter colours. Some concepts were introduced as relevant in the 2010s and seem to belong mostly to a single cluster, one which seems to be increasingly developing today.

**Fig. 11:**
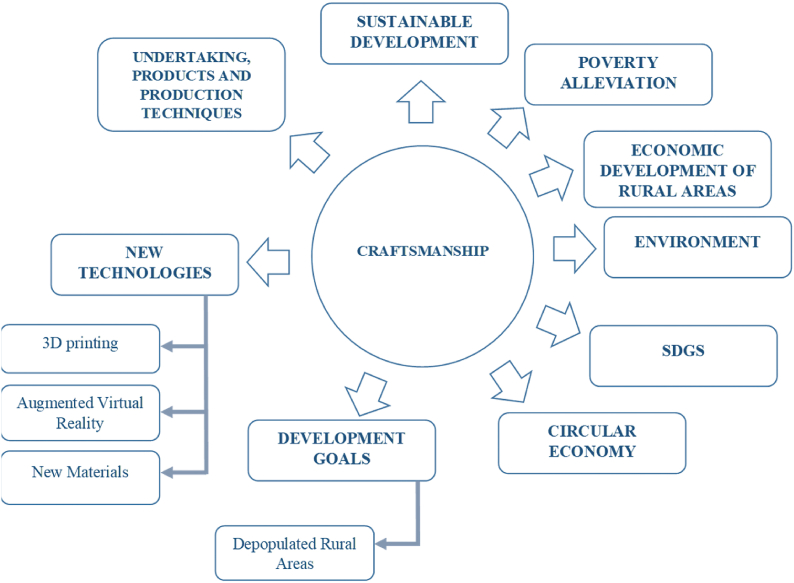
Future research lines.

Fig. 11 summarizes graphically the most relevant future lines of research in relation to the importance of crafts in sustainable rural development.

Next, the most relevant contributions of these lines of research and their evolution are developed, considering what is observed in Figs. 9 and 10.

##### 4.2.5.1 Cluster 1: Economic development of local communities in rural areas

Cluster 1: Economic development of local communities in rural areas.

The red cluster, consisting of 15 articles, is the largest of all and its keywords are found in 109 articles, which constitutes 10.55% of the total. These publications mainly deal with crafts aspects in rural areas as a source of income for the local community.

The main contributions that have been extracted from this cluster are as follows:

In the first place, handicrafts can be a very important source of income for local communities and they can be motivated by the development of ecoparks that promote handicrafts for ecotourism [10], by tourism in general, which in many cases leads to rural development and improved economic conditions ([13,14,61,62]), or by population moving to rural areas to improve their quality of life, as it is the case of small entrepreneurs seeking for quick profits in a rural niche [63]. In some cases, factors that affect the trade of craft products are analyzed ([64,65]).

On the other hand, tourism gives rise to numerous forms of crafts, such as those made from geological elements [10], craft products derived from other natural resources of the place [13], gastronomy as a cultural asset [14], wood crafts [62] or grass weaving ([61,62]).

Sustainability is another of the key concepts of this cluster, and it is one of the goals of the development of geoparks [10]. In some cases, regulation through policies that act in favor of sustainability is necessary when the trade of a resource used for crafts tends to deplete it, as in the case of various types of bark or wood ([61,66]) or non-timber forest products ([67,68]).

In the articles analyzed, the process of modernization of rural areas is also present [69]. However, seen from a negative point of view, one could say that “modernization can increase the insecurity of livelihoods” [62]. However, there are modern tools, such as mobile money, that have provided an advantage in trade for those local producers who have implemented them [65].

Employment promotion is another objective of ecotourism in geoparks [10], although in the crafts sector it is sometimes observed that recruitment of staff is often low [70] and that artisans seek job satisfaction not only in financial gain but also in doing their job well and having satisfied customers. The employment factor and economic benefit, or the search for a natural place to live, are variables that can be closely linked to migration to rural areas [10,70].

##### 4.2.5.2 Cluster 2: influence of crafts in rural areas

The second cluster is the green cluster, consisting of 13 items, found in a total of 98 research articles, and mainly deals with the changes that crafts or applications of new craft products or processes can bring about in rural areas, mainly at the economic and demographic levels. The main contributions are detailed below.

Many studies address the impact on the economy of the development of certain types of activities typical of the rural world, such as agriculture or, more conveniently, crafts. The revitalization of rural areas thanks to crafts can be seen [71]; o the positive effect of “indigenised” or indigenous handicrafts to give cultural value to the product and encourage the active participation of the local population [72], as well as the possibilities for economic development through non-timber products, which can lead to the creation of enterprises [73]. The improvement of artisanal processes in rural areas can also lead to an important improvement in economic, technical, and environmental terms [74]. Energy requirements of artisans in rural areas is analized by other studies [75] and try to improve living conditions by finding solutions to the problem of energy shortages [76].

Likewise, the improvement or deterioration of the economy can affect the number, specialisation and skill of artisans [77] and can lead to a sudden change of profession due to the development of a free port, which leads artisans to move on to other professions such as construction or transportation [78].

Regarding demographic changes, numerous reasons explain the increase of artisans and other occupations in rural areas, such as seeking isolation and nature in the countryside [62]. The timeframe is also important, since at the beginning of the 20th century the increase in population created employment opportunities for artisans [79], while at the end of the century the “rural exodus” had become a well-known phenomenon. However, there are cases of neo-rural phenomena, where a slight tendency to return to the rural sphere is observed in particular cases, particularly among artisans and smallholder farmers [80].

##### 4.2.5.3 Cluster 3: poverty in the rural world

The blue cluster is comprised of 8 items, which can be found in 82 search articles (7.9% of the total). It fundamentally deals with the aspect of poverty in the rural world. The main contributions are detailed below. Artisanal goods may be perceived by tourists as having a low value, as occurs in the Central American tropical forest, which means that their sale does not allow for an improvement in the quality of life of local residents and encourages them to deforest, as this provides more income than the sale of handicrafts and therefore leads to the loss of the richness of the forest [81].

Likewise, the population of rural areas specializing in handicrafts can be directly related to poverty in many areas, especially in the poorest parts of society. Some studies investigate the quality of the diet in these rural areas [82] and others that find an association between suffering from certain chronic diseases and work as artisans, temporary workers, housewives or small shopkeepers [83]. In the case of young children, there may also be a higher probability of malnutrition for children of artisans, peasants or small business women [84]. Some studies also analyze child labour in the artisanal sectors of certain regions [85].

While it is true that crafts may involve much more inefficient techniques than modern industry [85], in some cases, these crafts are fundamental for people's livelihood, as in South Africa [86]. In other cases, the economic progress of a specific place leads to a change of trade, as people move from crafts to other jobs [78].

Lastly, life in a rural area for artists and artisans is not always viable and this may depend, among other factors, on access to the market. This was the case in the study by Bunting and Mitchell, who identified the variables access to the market, landscape attractiveness and economic exigency as those affecting the location of artists and artisans in Canada [87].

## 5 Conclusions

Considering the research questions, this section develops the conclusions made and the implications of the findings.

As for the direction that research related to crafts and rural development has taken since the scientific community began to take an interest in this subject, it should be noted that starting from lines of research based on the analysis of the demographic and economic factors of development, research has progressed over the years towards more novel subjects such as sustainability, tourism development or care for the environment, as well as the prospects for employment and its improvement through the development of crafts.

Publications in this area address various aspects. Some deal with the impact of crafts and other activities in rural areas; the importance of small businesses for economic development, handicrafts, among others, or the analysis of the effects of handicrafts on the economy; others consider the factors that improve the development of craft activities in the rural world, such as attitude or technology. Lastly, other studies review the effects of goods such as the tropical forest, the native species of a region or gastronomic goods and their influence on the rural world and the artisan sector.

In addition, it is worth noting the high number of subject areas present in Fig. 4, as well as the weight of each of them. This demonstrates a high degree of multidisciplinarity in this area of knowledge.

Esteeming the number of citations of their articles, Rogerson, C.M., Braedt, O., Braunegg, G., Eyferth, J. and Dang, T.D., are the most important authors, among others. India, America, South Africa, Germany, Austria, Australia and Romania are the countries of origin of the ten authors with the most publications on the subject. Likewise, the countries with the highest number of citations are the United States, the United Kingdom, India, Canada and South Africa. Furthermore, the institutions with the most cited publications are the London School of Economics and Political Science (United Kingdom), the University of California (United States), the University of the Witwatersrand, Johannesburg (South Africa) and The University of British Columbia (Canada).

As for international cooperation, in the analysis of collaboration between the most relevant authors and institutions, little collaboration between them can be observed. However, some international collaboration is observed if we consider the set of publications from each country. The top countries for collaborating internationally on other countries' publications are the United States, Canada, China, Australia, the United Kingdom and India, while the top countries for collaborating with other countries on their own publications are the United Kingdom, South Africa, Canada, China, Italy and France. The field of research on crafts as a key factor in rural development has evolved from a perspective of economic and demographic factors towards other lines, such as sustainable development (so much in vogue nowadays), poverty alleviation, economic development of certain regions, impact on the environment and sustainability; in many cases directly linked to the development of tourism, which has developed exponentially in the last decades.

These are recurrent themes within the main publications in both the most prolific countries and the most cited publications, as can be seen in the analysis of the most important publications in each country, as well as in the most important keywords on this topic. Rural development is encouraged by governments around the world, with large investments that indicate their interest. Crafts have been a cleaner, more sustainable and environmentally friendly activity than industrial activities and constitute a source of possibilities and employment [17], linked above all to tourism [37]. In addition, crafts can achieve the best possible return on labour without harming the environment [19].

Studies related to crafts as a factor in the development of rural areas have grown significantly, prompted by the European Conference on Rural Development in 1996 [51] and by the economic crisis that led to the search for new sources of subsistence, in addition to the growing interest in more sustainable economic activities [22]. International collaboration in this field of study is generally scarce.

The most popular lines of research on this subject were, on the one hand, crafts as a source of income for local communities, especially linked to tourism, job creation and sustainability, and, on the other hand, the demographic and economic effects that the new products and craft processes have on the rural environment, followed by the alleviation of poverty in the rural world. This fundamentally highlights the concept of handicrafts as a source of subsistence for poor rural regions.

Crafts as an alternative or source of economic development and employment are part of rural development strategies. Some crafts are part of the development strategy of a region [88]. In sub-Saharan Africa, crafts and mining are a fundamental basis of the economy in rural areas ([15,89]). Specifically, artisanal and small-scale mining (ASM) is the most important non-agricultural activity in rural areas of sub-Saharan Africa [90]. Moreover, the Community-Based Natural Resource Management (CBNRM) programme in Botswana aims to achieve conservation and rural development and has generated new craft activities that provide an economic livelihood for the locals [13]. In the design of these strategies, crafts can play a relevant role in achieving goals. Thus, the Chilean government has developed policies that grant rights to artisanal fishermen and their goal is to improve the sustainability of the region [91]. The keyword analysis confirms this observation, as the terms “Developing country”, “Employment” or “Sustainable development”, as well as “Demography” or “Tourism” are among the 10 most frequently used keywords in this study. The most cited publications deal with topics such as improving access to marketing for small rural businesses, crafts as an important source of employment, solving the problems of rural exodus and the loss of craftsmen in order to preserve cultural heritage, or promoting craft activities vis-à-vis tourism, among others. This is because there are cases where crafts are a factor of interest and motivation for tourism, thus contributing to the development of rural areas.

Thus, ecotourism fosters the appearance of handicraft markets, and tourists show interest in touring the villages and visiting the handicraft markets, finding that many were willing to pay much higher rates than those proposed by the communities [92]. Another example of this is artisanal recipes. In many cases, the typical foods of numerous rural regions have been considered to have a clear impact on rural development [93].

Concerning the popularity of handicrafts in the rural world, it has been discovered that the relationship between rural development and handicrafts is addressed in rural development projects in various parts of the world. An example of this is the Community-Based Natural Resource Management (CBNRM) programme in Botswana [13] or rural development in West Wales, which analyses four sectors, one of them crafts [94]. Other studies analyze the contribution to regional development of craft beer companies in rural areas, specifically in 16 rural areas of Australia [38]. In Europe, LEADER actions are used to implement rural development strategies within the framework of the Common Agricultural Policy (CAP). These projects are based on the fact that crafts and agriculture “represent a sustainable example of human integration with nature” [29]. Thereby, in some cases and after decades of decline, crafts have experienced exponential growth in this century, due to social changes such as the interest in reducing gaps between rural and urban populations, or the taste for living in a rural area. This has contributed to the generation of social and symbolic capital, and financial, in those rural regions where crafts have been promoted [38], creating new connections between the rural world and the cities, with the aim of revitalizing rural businesses and communities through culture, art and crafts ([11,12]).

## 6 Limitations

To conclude, it is important to bear in mind that this research has certain limitations that should be considered for future research. Firstly, the database used for the study was Scopus and we consider that it would be necessary to use other repositories, such as Google Scholar, or Web of Science. Secondly, in order to facilitate comparison and understanding for certain analyses, only research articles have been taken into consideration. However, a greater diversity of documents could have been included in the study to complement the analyses carried out. In the third place, the VosViewer tool used for data visualization and grouping might yield different results if compared to other software tools. Finally, the bibliometric analysis methodology does not consider that citations require a specific time to be analyzed, so content analysis could complement the study to assess the quality of the research.

## 7 Future lines of research

Regarding future lines of research, and based on the results obtained by bibliometric analysis, it can be considered that, given the trend of the evolution of the previously mentioned keywords, it would be interesting to establish correlations between the concept of craftsmanship and the concepts of sustainable development, poverty alleviation, economic development of rural areas and care for the environment, as well as relating the subject of Crafts and SDGs, Crafts and Circular Economy. (See Fig. 11).

It would also be interesting to determine both the type and characteristics of entrepreneurship taking place in this sector and what products and production techniques are being used.

Another important line would be to determine how new technologies such as 3D printing, augmented virtual reality and new materials are relevant to the design and production of new craft-related products and services.

Likewise, as stated in this study's introduction, we should underscore the importance that these concepts have acquired in recent years and the interest on the part of governments in encouraging certain actions to achieve development goals in the future, particularly as solutions for what is called ‘emptied Spain’ (depopulated rural areas).

This type of research can provide relevant information to ensure those responsible for the design of public policies and the owners of handicraft companies can formulate strategies that would allow this productive sector to be sustainable in the coming years and adapt to the new realities demanded by the consumer and craft markets.

## Author contribution statement

All authors listed have significantly contributed to the development and the writing of this article.

## Funding statement

This study was funded by RRREMAKER. RRREMAKER MSC-RISE-H2020 project has received funding from the European Union’s Horizon 2020 Research and Innovation programme under the Marie Skłodowska-Curie, proposal number: 101008060.

## Data availability statement

Data will be made available on request.

## Declaration of interest’s statement

The authors declare no conflict of interest.
